# Assessment of Exposure to Mycotoxins in Spanish Children through the Analysis of Their Levels in Plasma Samples

**DOI:** 10.3390/toxins13020150

**Published:** 2021-02-15

**Authors:** Beatriz Arce-López, Elena Lizarraga, Reyes López de Mesa, Elena González-Peñas

**Affiliations:** 1 Department of Pharmaceutical Technology and Chemistry, Research Group MITOX, School of Pharmacy and Nutrition, Universidad de Navarra, 31008 Pamplona, Spain; barce@alumni.unav.es (B.A.-L.); mgpenas@unav.es (E.G.-P.); 2 Department of Pediatrics, Clínica Universidad de Navarra, Universidad de Navarra, 31008 Pamplona, Spain; rldemesa@unav.es

**Keywords:** child, digestive problems, human biomonitoring, mycotoxins, ochratoxin A, ochratoxin B, sterigmatocystin

## Abstract

In this study, we present, for the first time in Spain, the levels of 19 mycotoxins in plasma samples from healthy and sick children (digestive, autism spectrum (ASD), and attention deficit hyperactivity (ADHD) disorders) (*n* = 79, aged 2–16). The samples were analyzed by liquid chromatography-mass spectrometry (triple quadrupole) (LC-MS/MS). To detect Phase II metabolites, the samples were reanalyzed after pre-treatment with β-glucuronidase/arylsulfatase. The most prevalent mycotoxin was ochratoxin A (OTA) in all groups of children, before and after enzyme treatment. In healthy children, the incidence of OTA was 92.5% in both cases and higher than in sick children before (36.7% in digestive disorders, 50% in ASD, and 14.3% in ADHD) and also after the enzymatic treatment (76.6 % in digestive disorders, 50% in ASD, and 85.7% in ADHD). OTA levels increased in over 40% of healthy children after enzymatic treatment, and this increase in incidence and levels was also observed in all sick children. This suggests the presence of OTA conjugates in plasma. In addition, differences in OTA metabolism may be assumed. OTA levels are higher in healthy children, even after enzymatic treatment (mean OTA value for healthy children 3.29 ng/mL, 1.90 ng/mL for digestive disorders, 1.90 ng/mL for ASD, and 0.82 ng/mL for ADHD). Ochratoxin B appears only in the samples of healthy children with a low incidence (11.4%), always co-occurring with OTA. Sterigmatocystin (STER) was detected after enzymatic hydrolysis with a high incidence in all groups, especially in sick children (98.7% in healthy children and 100% in patients). This supports glucuronidation as a pathway for STER metabolism in children. Although other mycotoxins were studied (aflatoxins B1, B2, G1, G2, and M1; T-2 and HT-2 toxins; deoxynivalenol, deepoxy-deoxynivalenol, 3-acetyldeoxynivalenol, 15-acetyldeoxynivalenol; zearalenone; nivalenol; fusarenon-X; neosolaniol; and diacetoxyscirpenol), they were not detected either before or after enzymatic treatment in any of the groups of children. In conclusion, OTA and STER should be highly considered in the risk assessment of mycotoxins. Studies concerning their sources of exposure, toxicokinetics, and the relationship between plasma levels and toxic effects are of utmost importance in children.

## 1. Introduction

Children’s physiology differs from that of adults. Their higher metabolic rate, underdeveloped functional organs and relatively inefficient detoxification mechanisms make them more vulnerable to toxic compounds than adults [1]. Clinical symptoms and health outcomes can sometimes be more severe in children; in some cases, children are even affected by some compounds while adults are not. Moreover, due to their lower body mass, risk assessment should take into account the high internal dose to body weight ratio in this population group [2,3]. In addition, because of their longer life expectancy, children are prone to develop chronic syndromes in the future [3,4].

Children’s exposure to mycotoxins is not well known and has been linked to several acute and chronic pathologies [5,6,7]. Their symptoms and severity depend on the age, sex, immune system, and health status of the child and the specific toxins, or mixture of them, present in the diet, since ingestion is the main source of exposure to these toxic compounds [8,9]. Moreover, exposure by inhalation, skin, and mucous membranes, or a combination of two or more of these routes, should also be considered [1].

Acute mycotoxicoses are common in regions of developing countries in Africa and Asia, where the exposure of immunosuppressed children to certain mycotoxins is continuously high [10,11,12,13,14,15,16,17,18,19]. They have been associated with poor child growth and development, recurrent infections, immune suppression, and malnutrition [20,21,22]. Some authors suggest some relationships between impaired growth, kwashiorkor, or marasmus diseases and exposure to aflatoxins (AFs) and fumonisins (FBs) [19,23,24]. However, others explain that the presence of AFs in the tissues of children with these diseases is due to the lower metabolization of these toxins by damaged livers [1], and further studies are needed to clarify this aspect [25]. In addition, high ochratoxin A (OTA) levels in children’s blood have been related to microglobulinuria due to kidney damage [1].

On the other hand, the occurrence of symptoms associated with acute exposure to mycotoxins in immunocompetent children from industrialized countries has increased. In some cases, mycotoxicosis may appear without specific clinical manifestations, such as cough, nausea, vomiting, skin rash, etc., which could lead to an erroneous diagnosis. The most common acute pediatric effects of mycotoxins in developed countries are gastrointestinal diseases, when exposure to mycotoxins occurs through food or acute lung problems such as recurrent apnea or pneumonia, when exposure is due to inhalation or contact [26].

Despite all of the above, levels of mycotoxins found in the diet are often low, and chronic mycotoxicosis, associated with low-level exposure, can occur worldwide. Some long-term health effects (e.g., mycotoxin-related cancers) are rarely seen in children because of their long latency periods but are of great concern because they pose a significant risk to individuals exposed to carcinogenic mycotoxins [26]. Digestive disorders and neurological problems have also been linked to a chronic exposure to mycotoxins, but these relationships are not clear.

Mycotoxins can affect the digestive tract in two ways. The first is the alteration of the gut microbiota by exerting a toxic effect on the microbes, although this effect has been observed in studies using high concentrations of mycotoxins [27]. In addition, mycotoxins have been described as altering the structures of the intestine. Data available in a recent review show that dietary exposure to certain mycotoxins, especially trichothecene (TCT) and patulin (PAT), cause gastrointestinal problems because they affect the intestinal barrier, impairing the permeability and integrity of epithelial cells and causing inflammation of the mucosa. In this review, authors explain that human exposure to certain mycotoxins, in particular, deoxynivalenol (DON), can be related to the etiology of chronic inflammatory bowel diseases and to the prevalence of food allergies, particularly in children [28].

Other studies indicate an increased importance of diet, as the main route of mycotoxin exposure, in childhood neurobehavioral disorders [29]. They found that OTA inhibits some genes related to autism spectrum disorder (ASD) with a gender-specific toxicity for men. However, more research needs to be done to explain the role of OTA in symptoms related to neurodevelopmental disorders [30], and controversial data have been observed. Although De Santis et al. (2017, 2019) [29,30] found a significant association between OTA in children with ASD compared to healthy children, Duringer et al. (2016) did not find this association [31].

In summary, the extent and severity of children’s exposure to mycotoxins needs to be evaluated [4]. In this assessment, biological differences between children and adults need to be taken into account. It is also necessary to increase the knowledge of the symptoms that can be caused by mycotoxins in order to detect the mycotoxicosis, apply adequate treatments, and protect the health of children [26].

Exposure assessment to mycotoxins in children could be done by conducting studies on the presence of mycotoxins in food (external exposure) and by biomonitoring the presence of mycotoxins in biological samples (internal exposure).

Traditionally, the first approach has been used, although few studies have been carried out to evaluate food for children. Raiola et al. (2015) reviewed the studies conducted to understand the occurring mycotoxins in food for children. These authors concluded that more restrictive limits of mycotoxins in food for children are needed [3]. More recently, Assunção et al. (2018) [32] concluded that Portuguese children’s food was contaminated with several mycotoxins, especially by AFs. Another study of these authors showed that the co-occurrence of PAT and OTA in food samples in Portugal could have a major impact on intestinal health [33]. Other authors found low levels of different mycotoxins in children’s foods [34,35,36], but all of them suggest studies for food control, especially to reduce the exposure of children to OTA, AFs, and TCT. In general, cereal-based products are of great importance in terms of mycotoxin exposure as they are especially consumed by children [37] and were the most contaminated by these toxic compounds, especially zearalenone (ZEA), FBs, OTA, and deoxynivalenol (DON) [34]. Maize is considered one of the most dangerous ingredients among cereals, in particular because of the eating habits or specific pathologies of vulnerable groups, such as those with celiac disease, due to the large consumption of corn products [36]. In a study from Tunisia, Oueslati et al. (2014) [38] evidenced a high prevalence of several mycotoxins (including aflatoxin B1 (AFB1), OTA, and sterigmatocystin (STER)) in sorghum, a product widely consumed in the diet of children and infants. Moreover, sorghum is a potential alternative food for the celiac population, and therefore, special attention should be focused on these vulnerable groups. In Spain, exposure rates obtained for PAT and OTA, among other mycotoxins, in fruit juices, evidenced an increasing risk for children through their consumption [39].

The internal exposure approach, that is, the analysis of biomarkers of exposure in biological matrices, is complementary to the analysis of mycotoxins in food and it presents some advantages: no need to identify the source of contamination, no problems related to sampling and food analysis or collection of consumption data in a varied diet and no dependence on the preparation process or the bioavailability and biological capacity of the food [37,40]. Few studies have applied the internal exposure approach to assess the risk of mycotoxins to children’s health, and most of them are devoted to the exposure during the very first months or years of life [11,12,14].

The current work aims to present the results obtained in the biomonitoring of mycotoxins and their conjugates in plasma from Spanish children aged 2 to 16 years. To the best of our knowledge, this is the first study in Spain evaluating the exposure to 19 mycotoxins in plasma samples from healthy children. Children with digestive problems, ASD, or attention deficit hyperactivity disorder (ADHD) were also included in the study.

Another interesting point of this study is that the obtained results can be compared with those described in a recent similar study in adult plasma samples in Spain, in which the same methodology was applied and the same compounds were analyzed [41].

## 2. Results and Discussion

### 2.1. Control of the Analytical Sequences

Matrix-matched calibration curves were prepared for each mycotoxin from calibrators analyzed along with the samples. All calibration curves satisfied the criteria previously defined: a minimum of six points, R^2^ > 0.99, and a back-calculated mycotoxin concentration for each calibrator not differing by more than 15% from the nominal value (20% for limit of quantification (LOQ)). Examples of the obtained calibration curves before and after enzymatic treatment are presented in Supplementary Materials Table S1. Furthermore, qualification (q) and quantification (Q) transitions were presented for each mycotoxin detected and the corresponding q/Q ratio (in %) did not differ by more than 20% from the mean q/Q ratio obtained for this mycotoxin in calibrators (Table 1). Finally, retention times (RT) of the detected mycotoxin in samples did not differ from the mean value in the calibrators by more than 1.5% for OTA before and 1.3% after the enzymatic treatment. In the case of ochratoxin B (OTB), the differences were no more than 0.7% and 0.3%, respectively. For STER they were not higher than 0.5% (Table 1).

### 2.2. Plasma Samples

Seventy-nine samples were obtained from 79 different donors. Nearly 50% of the samples (*n* = 40) were from healthy volunteers (28 girls and 12 boys); while 39 were from patients with different pathologies: two samples were from boys with ASD, 7 from children with ADHD (6 boys and 1 girl), and 30 from sufferers of digestive disorders (15 boys and 15 girls).

Participants were between 2 and 16 years old. Healthy children (mean age of 10.6 ± 2.8 years) were slightly older than patients (9.0 ± 3.9 years) (*p* = 0.044, 95% confidence interval (CI). The global mean age was 9.8 ± 3.5 years. In Table 2, the distribution of donors according to health status, gender, and age is shown.

### 2.3. Chromatographic Results

**Figure 1:**
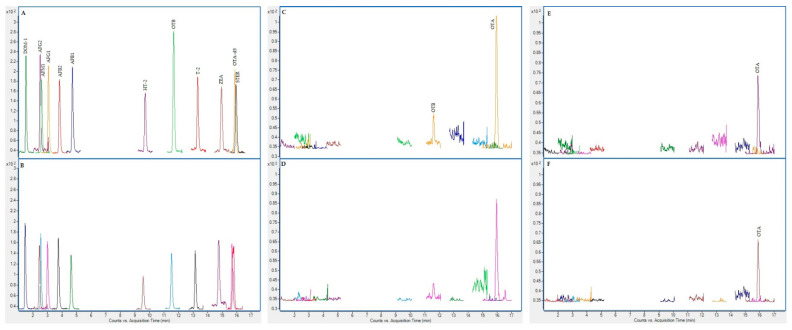
Superposed extracted chromatograms obtained for mycotoxin group I from a calibrator at 10× limit of quantification (LOQ) level (**A**,**B**) and plasma samples from a healthy child (PS37) (**C**,**D**) and a patient child (PD23) (**E**,**F**) before enzymatic treatment. (**A**,**C**,**E**) display the Q transition, and (**B**,**D**,**F**) the q transition, respectively.

**Figure 2:**
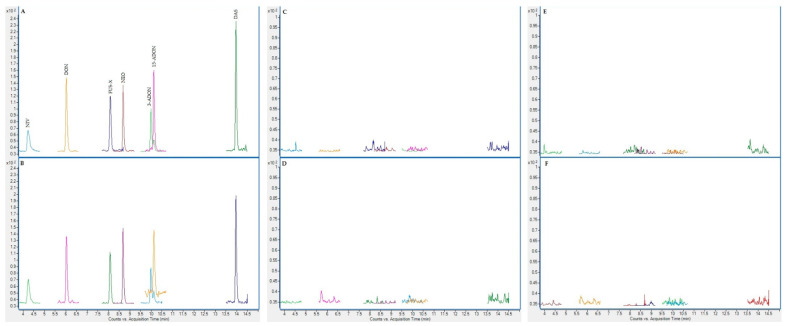
Superposed extracted chromatograms obtained for mycotoxin group II from a calibrator at 10× LOQ level (**A**,**B**) and plasma samples from a healthy child (PS37) (**C**,**D**) and a patient child (PD23) (**E**,**F**) before enzymatic treatment. (**A**,**C**,**E**) display the Q transition, and (**B**,**D**,**F**) the q transition, respectively.

**Figure 3:**
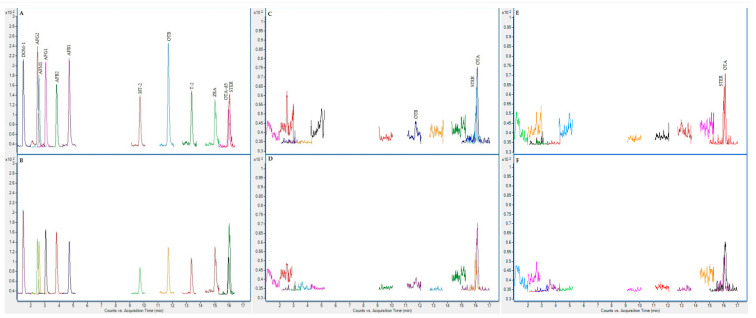
Superposed extracted chromatograms obtained for mycotoxin group I from a calibrator at 10× LOQ level (**A**,**B**) and plasma samples from a healthy child (PS37) (**C**,**D**) and a patient child (PD23) (**E**,**F**) after enzymatic treatment. (**A**,**C**,**E**) display the Q transition, and (**B**,**D**,**F**) the q transition, respectively.

**Figure 4:**
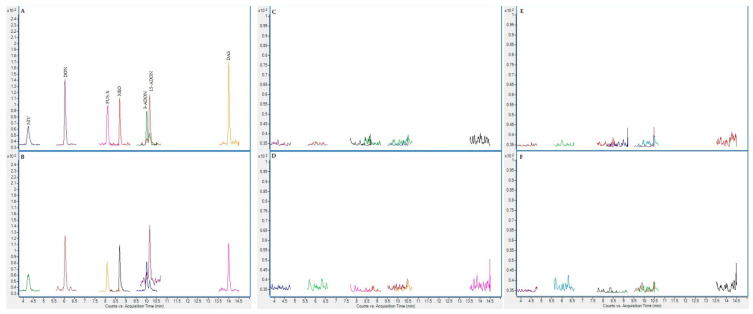
Superposed extracted chromatograms obtained for mycotoxin group II from a calibrator at 10× LOQ level (**A**,**B**) and plasma samples from a healthy child (PS37) (**C**,**D**) and a patient child (PD23) (**E**,**F**) after enzymatic treatment. (**A**,**C**,**E**) display the Q transition, and (**B**,**D**,**F**) the q transition, respectively.

On the following figures (Figure 1, Figure 2, Figure 3 and Figure 4), superposed extracted chromatograms obtained from calibrators and samples are shown. Figure 1 and Figure 2 represent chromatograms before enzymatic treatment for healthy and patient children and for group I and II mycotoxins, respectively. Likewise, Figure 3 and Figure 4 represent samples after enzymatic treatment. Moreover, for each extracted chromatogram, both transitions (Q and q) are displayed.

### 2.4. Mycotoxins in Samples

**Figure 5:**
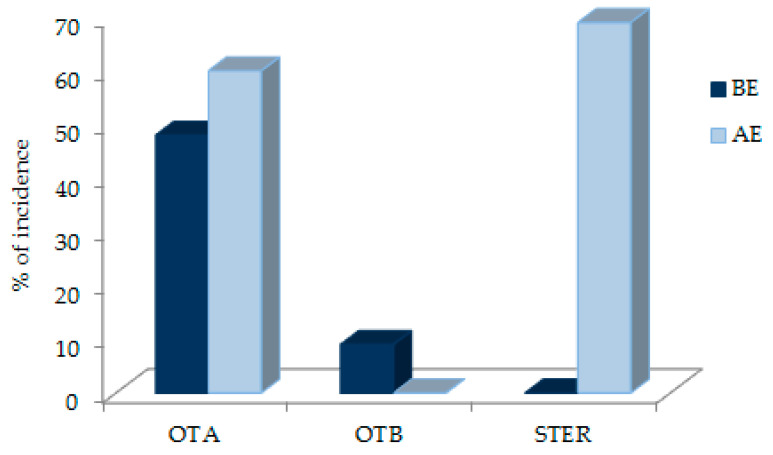
Global incidence (%) of each of the obtained mycotoxins (OTA, OTB, and STER) according to enzymatic treatment. AE: after enzymatic treatment. BE: before enzymatic treatment.

Results of the detected mycotoxins (OTA, OTB, and STER) obtained for each one of the samples before and after enzymatic treatment of healthy and patient children are shown in Table 3 and Figure 5.

Data obtained for OTA and STER, before and after enzymatic treatment, are summed-up in Table 4 and Table 5.

Only those groups with a significant number of samples were included in the statistical analysis: samples of healthy children (*n* = 40) and children with digestive problems (*n* = 30). Data from children with ASD (*n* = 2) or ADHD (*n* = 7) were not included in the statistical analysis due to the low number of samples, although the obtained values in these samples were also discussed. The highest OTA value (34.2 ng/mL) was considered an outlier and was not included in the statistical comparison. 

Regarding OTA and gender, although the mean OTA level was slightly higher in girls than in boys, the difference did not reach statistical significance in either group, healthy children or children with digestive disorders, either before or after the enzyme treatment (*p* > 0.05, 95% CI). Therefore, data for girls and boys were considered a unique group for comparison between healthy children and those with digestive disorders. The statistical *p*-values obtained in the different comparisons in terms of OTA incidence are shown in Supplementary Materials Table S2.

#### 2.4.1. Results before Enzymatic Treatment

The analysis of the children’s plasma samples showed the presence of OTA and OTB. The other studied mycotoxins: deepoxy-deoxynivalenol (DOM-1), aflatoxin G2 (AFG2), aflatoxin M1 (AFM1), aflatoxin G1 (AFG1), aflatoxin B2 (AFB2), AFB1, ZEA, STER, T-2 toxin (T-2), HT-2 toxin (HT-2), DON, fusarenon-X (FUS-X), neosolaniol (NEO), 3-acetyldeoxynivalenol (3-ADON), 15-acetyldeoxynivalenol (15-ADON), or diacetoxyscirpenol (DAS) were not found in any of the analyzed samples at levels higher than the limits of detection (LODs) of the method.

**Figure 6:**
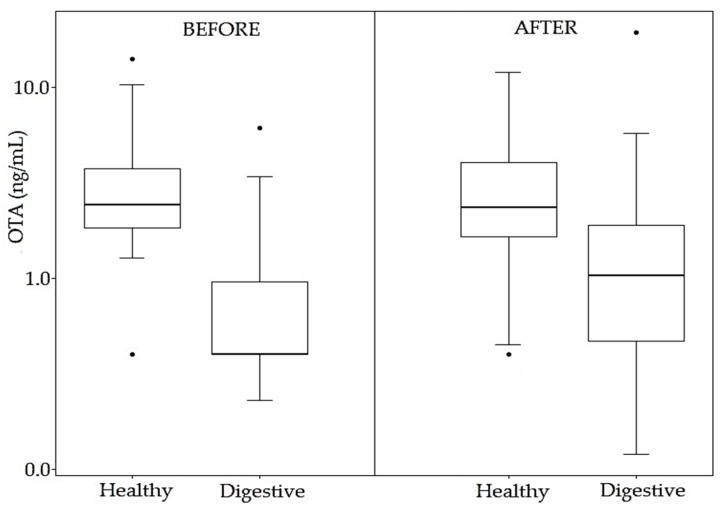
Comparison of OTA incidence before and after enzymatic treatment in healthy children and children with digestive disorders.

OTA was the most prevalent mycotoxin (*n* = 50/79) (63.3%), and this prevalence was in all groups, with >LOD levels in 92.5% (*n* = 37/40) of healthy children, 36.7% (*n* = 11/30) of children with digestive disorders, 50% (*n* = 1/2) of patients with ASD, and 14.3% (*n* = 1/7) of patients with ADHD. The incidence of OTA is much higher in healthy children than in children with digestive disorders (Figure 6). In addition, the lowest incidence of OTA is in patients with ADHD, although few samples were collected from these groups of patients.

In other studies in children, OTA was also the most prevalent mycotoxin in the analyzed plasma or serum samples. De Santis et al. (2017) [29] found levels of OTA at 82.9% in serum samples from 233 Italian children (aged 2–12 years); these authors also found other mycotoxins, but with a lower incidence: AFB1 (22.9%), AFM1 (50.2%), ZEA (5.4%), DON (19.5%), and DOM-1 (13.1%). Warensjö et al. (2020) after studying the presence in the serum of 27 mycotoxins and related compounds, including AFs, DON, T-2, HT-2, OTA, and ZEA, among others, in more than 1000 school students in Sweden (11–18 years old) detected OTA in 100% of the serum samples, while among the other analyzed compounds, and apart from OTA, only 2’R-OTA and enniatin B were found [42]. Moreover, in the work of Erkekoǧlu et al. (2010) [43], the incidence of serum OTA in children was 100%.

In a recent work published by our group [41], the same 19 compounds were analyzed in plasma samples from Spanish adults. OTA was also the most prevalent mycotoxin (97.3%) with a mean value of 2.87 ng/mL and a concentration range of <LOD to 19.9 ng/mL, similar to the values obtained in healthy children from the same country.

Therefore, studies on sources of exposure, bioaccessibility, bioavailability, toxicokinetics, and risk assessment of this mycotoxin are of utmost importance in children and adults.

In the work of De Santis et al. (2017) [29], samples of 172 autistic and 61 healthy children were analyzed. The incidence of OTA in healthy children was 76.4% with a mean value of 0.27 ng/mL; while in autistic children, it was 85.1% with a mean value of 0.39 ng/mL, and the authors indicate significant differences in the means between both groups. In the present study, the incidence of OTA in children with ASD was 50% (1.1 ng/mL), but only two samples were available.

OTA levels ranged from <LOD (0.4 ng/mL) to 14.1 ng/mL. However, one sample reached the highest value of 34.2 ng/mL, which corresponds to a celiac girl patient (PD3) (Table 3).

The mean OTA concentration in healthy subjects was much higher (3.21 ng/mL) (range <LOD–14.07 ng/mL) than that in children with digestive disorders 0.99 ng/mL (<LOD to 6.07 ng/mL) (Table 4). Significant differences were observed when comparing both groups using a Wilcoxon test (*p* < 0.05, 95% CI) (Supplementary Materials Table S2).

OTB, a toxin not usually included in biomonitoring studies of mycotoxins, appeared only in a low percentage of healthy child samples (9 samples) (11.4%), with levels in the range of 0.4 to 0.8 ng/mL and a mean value of 0.57 ng/mL. All the samples that showed positive levels of OTB (>LOD), also presented OTA levels. In a previous work of our research group in which OTB was biomonitored in adult plasma, the incidence of OTB was 10% with a mean of 0.51 ng/mL [41], very similar results to those obtained in healthy children. Nevertheless, this mycotoxin was not found in children with digestive disorders, ASD, or ADHD. In the paper of Arce-López et al. (2020) [41], the authors related the presence of OTB to the human OTA metabolism. 

#### 2.4.2. Results after Enzymatic Treatment

After enzymatic treatment, 67 plasma samples contained OTA (>0.4 ng/mL), 37 and 23 of them were from healthy children and children with digestive disorders, respectively (Table 3). Therefore, the total incidence of OTA was 92.5% and 76.6%, respectively. In addition, 85.7% (6/7) of the incidence was observed in the ADHD group.

Therefore, the incidence of OTA in healthy children after conjugated hydrolysis remained high and similar to that obtained before enzymatic treatment and no significant differences were observed before and after the treatment (*p* > 0.05, 95% CI) (Figure 6). However, in 42.5% of individuals, there was an increase in OTA levels after hydrolysis. This indicates the presence of OTA conjugates in plasma samples from healthy children.

A general increase of incidence after enzymatic treatment was observed in the patient groups. In the group of patients with digestive disorders, it was from 36.7% to 76.7% (Table 4), and significant differences were observed before and after treatment (*p* < 0.05, 95% CI) (Figure 6). In the group with ADHD, the incidence also increased from 14.3% to 85.7%. In the ASD group, the incidence of OTA remained stable, but OTA levels increased, although only two samples were available. Because the enzymatic treatment indicates the presence of OTA conjugates in the plasma samples, these results could once again indicate differences in OTA metabolism between healthy children and patients.

The mean OTA concentration level was higher in healthy children (3.29 ng/mL) than in digestive patients (1.90 ng/mL) (Table 4), with levels in the range of <LOD to 11.9 ng/mL (healthy) (19.3 ng/mL digestive disorders). Significant differences were observed between the two groups (*p* < 0.05, 95% CI) (Supplementary Materials Table S2). In the ADHD and ASD groups, mean OTA levels were also lower than in the healthy group (0.82 and 1.90 ng/mL), respectively.

After enzymatic hydrolysis, OTB did not appear in either healthy children or patients.

**Figure 7:**
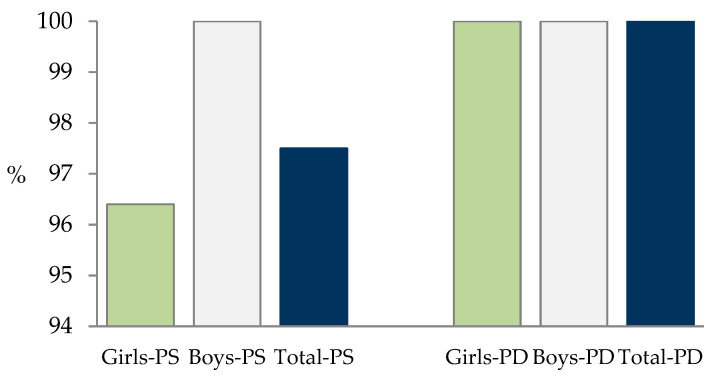
Percentage of STER incidence after the treatment. PS: healthy children. PD: children with digestive disorders.

As shown in Table 5, STER appeared in almost all the samples after the treatment with the mixture of enzymes, and no significant gender differences were found in either group (healthy or digestive patients). STER was found in 78 plasma samples (98.7%), 39 from healthy children (95.1%), 30 from digestive patients (100%), and also in 100% of the samples from the ASD and ADHD groups. The incidence of this mycotoxin is high in all groups, especially in patients (Figure 7), and was also high in adults in Spain (85.8%) [41].

STER appeared in the range of 0.9 to 2.5 ng/mL and the mean total levels of STER were 1.41, 1.53, 1.45, and 1.27 ng/mL for healthy, children with digestive disorders, ASD, and ADHD, respectively (Table 3). Patients with digestive disorders have higher mean and median values than healthy children, although no significant differences have been observed between the levels of both groups (*p* > 0.05, 95% CI). These results support, on the one hand, conjugation as a route for human metabolization of this mycotoxin and, on the other, the exposure to STER of adults and children in Spain.

Very few studies have been conducted on STER presence in food, but levels of this toxin have been found in cheese, spices, cereals, rice, sorghum, peanut paste, nuts, soybean, and beer; and, in some samples, in combination with AFs. It has also been found in indoor air and in dust from buildings contaminated with fungi [44].

The toxic effects of STER have been reviewed by Díaz Nieto et al. (2018) [44], and this mycotoxin has been classified as a possible human carcinogen (group 2B) by the International Agency for Research on Cancer (IARC) [45].

Therefore, due to its toxicity and presence in plasma from children and adults, this mycotoxin should be considered in the risk assessment for mycotoxins.

Finally, and after comparing the results in healthy children obtained in this study with those encountered recently in adults in Spain [41], after the analysis of the same 19 compounds in plasma samples using the same analytical method, the same compounds have been observed in both groups of donors. In the plasma samples of both studies, OTA and OTB have been found; STER was detected, but only after enzymatic treatment; and the other 16 mycotoxins studied were not detected in any sample.

## 3. Conclusions

We present here, for the first time in Spain, the exposure of children to multiple (19) mycotoxins through the analysis of plasma samples. Plasma samples came from children aged between 2 and 16 years and with different health statuses. Forty samples were from healthy children, and 39 from children suffering from digestive disorders and ASD or ADHD disorders.

Before enzymatic treatment, OTA and OTB were detected in the plasma analysis of the children and any other mycotoxin among those analyzed—DOM-1, AFG2, AFM1, AFG1, AFB2, AFB1, ZEA, STER, T-2, HT-2, DON, FUS-X, NEO, 3-ADON, 15-ADON or DAS—was not found at levels higher than the LODs of the method in any of the samples.

According to this and other studies conducted on plasma from children and adults, OTA was the most prevalent mycotoxin and was in a concentration range of >LOD to 34.3 ng/mL in the analyzed samples. OTA was more prevalent (and with higher levels) in healthy children’s plasma than in that of sick children.

OTB appeared only in those samples from healthy children with a low incidence and always co-occurring with OTA, results very similar to those obtained in adults in Spain. This mycotoxin was not found in children with digestive disorders, ASD, or ADHD.

After enzymatic treatment, the incidence of OTA in healthy children remained high and similar to that obtained before enzymatic treatment. However, according to data obtained in adults, in a percentage of individuals’ OTA levels increased, suggesting the presence of OTA conjugates in plasma samples from healthy children. It is remarkable that, in all patient groups, the incidence and levels of OTA increased after enzymatic treatment.

STER was detected in almost all samples, but only after treatment with the enzyme mixture, similar to the way this mycotoxin was detected in adults. These results support glucuronidation as a metabolism pathway in children for this toxin. Moreover, this mycotoxin presents high incidence, especially in the three groups of sick children, for whom it was 100%.

In conclusion, it appears that the exposure of healthy adults and children to mycotoxins is similar in this region of Spain. In the case of children with digestive disorders and, also, for ASD and ADHD, the same mycotoxins have been found, but their levels, incidence and also their behavior after β-glucuronidase/arylsulfatase treatment is somewhat different. These results may indicate differences in OTA metabolism between groups of healthy children and patients.

OTA and STER should be highly considered in the risk assessment for mycotoxins. Studies concerning their sources of exposure, toxicokinetics, and the relationship between plasma levels and toxic effects are of utmost importance in both children and adults.

## 4. Materials and Methods

### 4.1. Subject Recruitment

Donors were healthy children (*n* = 40) and children with different pathologies (digestive disorders (*n* = 30), ADHD (*n* = 7), and ASD (*n* = 2)) and were selected from the Department of Pediatrics of the Clínica Universidad de Navarra. Among the patients with digestive disorders, there were 11 celiacs, 10 with fructose/lactose intolerance, 2 had eosinophilic esophagitis, 1 ulcerative colitis, 2 *Helicobacter*-associated gastritis, and 4 chronic abdominal pain. Written informed consent was obtained from all of them for their participation, and the procedure was approved by the Ethical Committee of the University of Navarra (project 2018.193) on 10 April 2019. All the children included in this study were under 16 years of age, thus the legal guardian/s provided the informed consent for their participation. Blood samples (*n* = 79) were obtained during September 2019 and March 2020. Unfortunately, the pandemic situation due to COVID-19 limited sample collection, especially for sick children. Participants only gave their gender and age as personal information.

### 4.2. Plasma Sample Collection

Each volunteer gave 5 mL of blood, which was collected in BD Vacutainer^®^ Plasma Tubes (Madrid, Spain) using EDTA as an anticoagulant. Each tube was centrifuged at 12,000× *g* for 10 min at 4 °C, then plasma was frozen and stored at −80 °C until analysis.

### 4.3. Sample Analysis

The 79 samples were analyzed for mycotoxin presence before and after enzymatic treatment. Mycotoxins were analyzed using an LC system 1200 series coupled to a 6410 Triple Quadrupole (QqQ) in ESI (+) mode (Agilent Technologies, Waldbronn, Germany). The methodology was that described by Arce-López et al. (2020) [46], and the chromatographic parameters are summed-up in Table 6.

Using this methodology, 19 compounds (mycotoxins and metabolites) can be quantified. Depending on the physicochemical characteristics of the compounds, they were divided into two groups, and each one needed a different elution program for the chromatographic separation. DOM-1, AFG2, AFM1, AFG1, AFB2, AFB1, OTB, ZEA, STER, OTA, T-2, and HT-2 were included in group I. Nivalenol (NIV), DON, FUS-X, NEO, 3-ADON, 15-ADON, and DAS were included in group II.

The reagents used were as follows: deionized water (>18 MΩcm^−1^ resistivity) from an Ultramatic Type I system (Navarra, Spain), methanol (LC-MS grade) from Honeywell Riedel-de Haën (Seelze, Germany), acetonitrile (ACN) (HPLC grade) from Merck (Darmstadt, Germany), and formic acid (MS grade, purity >98%) and ammonium formate (MS grade) from Fluka Sigma-Aldrich (Mannheim, Germany). All mycotoxins and ochratoxin A-(*phenil*-d_5_) (OTA-d_5_) were obtained from Sigma-Aldrich (St. Louis, MO, USA) (reference material, purity ≥98%) as solutions in acetonitrile. Mixed stock solutions containing the mycotoxin standards (group I and II) were prepared by taking the appropriate volume from each individual standard solution, then diluted in ACN and stored at −20 °C until analysis. OTA-d_5_ was used in the preparation of calibrators instead of OTA. Due to their toxicity, a face shield and gloves were used when handling spiked samples.

Calibration samples were prepared by spiking human plasma. Different volumes of a mixed stock solution of mycotoxins were poured into 15 mL polypropylene centrifuge tubes and dried in an evaporator (GeneVac, SP Scientific, Ipswich, England) under vacuum at 60 °C. The residue was reconstituted using 15 µL of ACN and 450 µL of human plasma. The method for plasma treatment, before and after enzymatic treatment, was described in Arce-López et al. (2020) [46]. Concisely, it was as follows: 0.4 mL of human plasma was passed through a Captiva EMR-lipid cartridge that contained 1.2 mL of acetonitrile (1% formic acid). The eluate was divided into two 0.4 mL portions that were evaporated until dry (60 °C). The residue resulting from one portion was reconstituted with 200 µL of 40% B-mobile phase for analyzing mycotoxins group I. The other one was reconstituted with 200 µL of 5% B-mobile phase for analyzing mycotoxins group II. The presence of Phase II metabolites in samples was assessed, reanalyzing the samples after enzymatic treatment with β-glucuronidase/arylsulfatase (from Helix Pomatia, Sigma Aldrich, Mannheim, Germany). For this purpose, 50 µL of β-glucuronidase/arylsulfatase enzyme (250 U/mL, 0.2 U/mL in phosphate buffer solution (PBS)) was added to 400 µL of plasma, and samples were maintained at 37 °C (water bath) overnight. Then, they were processed as described above.

This methodology (before and after enzymatic treatment) was successfully validated following the Food and Drug Administration (FDA) and European Medicines Agency (EMA) guidelines for bioanalytical method validation following the procedure described in Arce-López et al. (2020) [46]. The resulting LOD values were as follows: 1.35 ng/mL for DOM-1; 0.35 ng/mL for AFG2, 0.18 ng/mL for AFM1; 0.07 ng/mL for AFG1 and AFB2; 0.04 ng/mL for AFB1; 2.70 ng/mL for HT-2; 0.40 ng/mL for OTA and OTB; 0.20 ng/mL for T-2 and STER; 1.80 ng/mL for ZEA; 9.10 ng/mL for NIV; 1.94 ng/mL for DON; 1.95 ng/mL for FUS-X; 0.18 ng/mL for NEO; 0.70 ng/mL for 3-ADON; 1.20 ng/mL for 15-ADON; and 0.15 ng/mL for DAS. Recoveries values (studied in intermediate conditions at three concentration levels) were 68.8% for STER; 77.8% for OTA-d_5_; 81.6% for ZEA and NIV; 82.4% for AFB1; 82.7% for AFG1; 83.2% for OTB; 85.0% for AFB2; 88.2% for AFM1; 89.7% for DON; 89.8% for HT-2 and DOM-1; 90.1% for AFG2; 91.2% for T-2; 91.4% for FUS-X; 92.6% for 15-ADON; 93.5% for NEO; 95.1% for 3-ADON; and 97.6% for DAS (RSD ≤ 15% for all the mycotoxins).

### 4.4. Control of the Analytical Sequences

For analysis control, at least eight matrix-matched calibrators were analyzed along with the samples in each one of the analytical sequences. These calibrators were employed to obtain the calibration curves used for mycotoxin quantification. The criteria that should be accomplished for calibration curves were as follows: a minimum of six points, a determination coefficient (R^2^) > 0.99, and a back-calculated concentration for calibration samples not different (relative error of the mean (RE) in %) by more than 15% from the nominal value (20% for LOQ level) [47].

The identification of each mycotoxin in samples was carried out based on the presence of both, q and Q, product ions in the chromatogram with a ratio (q/Q in %) that did not differ more than 20% from the obtained mean ratio in calibrators of the corresponding sequence. Besides, RTs should not differ by more than 2.5% from the mean of the RTs for each mycotoxin in the calibrators [48].

### 4.5. Statistical Analysis

Data were not normally distributed (Shapiro–Wilk test), hence equal variance was not assumed. Non-parametric tests were performed to investigate possible associations or differences between the groups. A Wilcoxon rank sum (Mann–Whitney) was used to study differences between each group (healthy and patients) and gender (boys and girls). Differences due to the enzymatic treatment (before and after treatment) were analyzed by a Wilcoxon signed-rank test.

Data above the corresponding LOD were included in the statistical analysis, whereas the LOD/2 value was used for data below LOD. All the analyses were performed using RStudio version 1.2.5019 (Boston, MA, USA). Statistical significance was set at *p*-value < 0.05 (95% CI).

## Figures and Tables

**Table 1:** Qualification/quantification (q/Q) ratios (%) and retention times (RTs) for calibrators and samples for ochratoxin A (OTA), ochratoxin B (OTB), and sterigmatocystin (STER).

		OTA		OTB		STER
		BE	AE	BE	AE	AE
Calibrators	q/Q ratio	77.41 ± 4.09	78.50 ± 5.71	44.74 ± 2.23	46.02 ± 1.45	95.07 ± 12.87
	RT (min)	15.90 ± 0.22	16.04 ± 0.07	11.68 ± 0.17	11.79 ± 0.06	16.10 ± 0.07
Samples	q/Q ratio	73.69 ± 7.63	78.94 ± 9.22	43.65 ± 3.99	46.06 ± 6.35	97.17 ± 12.15
	RT (min)	15.96 ± 0.13	16.18 ± 0.06	11.60 ± 0.08	11.74 ± 0.04	16.10 ± 0.06

AE: after enzymatic treatment; BE: before enzymatic treatment.

**Table 2:** Distribution of donors according to their health status, gender and age.

Health Status	Gender	*n*	Range (Years)	Mean ± SD (Years)	Total Mean ± SD (Years)
Healthy	Girls	28	6–15	11.2 ± 2.4	10.6 ± 2.8
	Boys	12	4–13	9.1 ± 3.1	
ADHD	Girls	1	6	6	9.7 ± 3.3
	Boys	6	7–14	10.3 ± 3.1	
ASD	Girls	0	n.s.	n.s.	6.5 ± 2.1
	Boys	2	5–8	6.5 ± 2.1	
Digestive	Girls	15	2–16	8.1 ± 4.4	9.0 ± 4.4
	Boys	15	4–15	10.3 ± 3.9	

n.s.: no sample. ADHD: attention deficit hyperactivity disorder, ASD: autism spectrum disorder. SD: standard deviation.

**Table 3:** Levels of OTA, OTB, and STER (ng/mL) found in healthy and patient children’s plasma samples.

Healthy Girls		BE		AE		Healthy Boys		BE		AE		Patient Girls		BE	AE		Patient Boys		BE	AE	
Sample	Age	OTA	OTB	OTA	STER	Sample	Age	OTA	OTB	OTA	STER	Sample	Age	OTA	OTA	STER	Sample	Age	OTA	OTA	STER
PS2	13			2.9	1.6	PS1	12	4.7	*0.5 ^a^*	*1.9*	1.2	PD2	16	2.9		1.6	PD1	12			1.2
PS3	11	2.3		*1.2*	1.5	PS5	4	4		4.7	2.5	PD3	4	34.2	19.3	1.7	PD5	6	*0.5*	3.4	2.1
PS4	10	2.8		*1.9*	1.3	PS6	6	6		2.6	*0.9*	PD4	6	*0.5*	*0.7*	1	PD6	8		0.5	1.3
PS7	9			2.1	1.5	PS9	13	2.6	*0.4*	2.3	1.1	PD9	14	2	*1.9*	1.6	PD7	15		1.1	1.4
PS8	12	*1.5*		3.8	1.2	PS15	10	2.7		*1.6*	1.2	PD10	3		*0.5*	1.9	PD8	15		1.9	1.3
PS10	12	*1.5*			1.4	PS23	11	2.4		2.9	1.5	PD12	13	2.2	2.9	1.8	PD11	8		1.8	1.8
PS11	6	3.9	*0.7*	11.9	1.6	PS25	10	*1.8*		2	1.3	PD13	7		2.2	1.8	PD15	5		1.2	1.7
PS12	10	*1.9*		*1.9*	2.5	PS27	7	2.3		4	2	PD14	9		*1.5*	1.4	PD16	7		0.9	1.9
PS13	12	3.9		2.6	1.6	PS28	9	4.3	*0.5*	4.9	1	PD17	9		*1.3*	1.6	PD18	5		1.3	2.4
PS14	13			*1.6*	1.6	PS31	4	2.4	*0.5*	2	1.3	PD19	9		*0.7*	1.6	PD20	13			*0.8*
PS16	15	3.4		4.1	1.5	PS39	12	*1.3*		2.5	1.3	PD22	9	3.4		1.5	PD21	12	*1*		1.1
PS17	8	*1.7*		*1.7*	1.4	PS40	11	2.4		2.5	1.3	PD24	3			1.5	PD23	10	6.1	5.8	1.5
PS18	9	2.4		2.1	1.2							PD25	5		*0.8*	1.3	PD26	4		0.7	1.6
PS19	11	*1.9*		*1.2*	1.5							PD28	2	*1.3*	*1.4*	1.4	PD27	12		2.2	1.9
PS21	12	5.3		9.1	1.6							PD29	13		*0.6*	1	PD30	15	*1.6*		1.4
PS22	14	2.2		7	1.4												PA1	5			1.4
PS24	14	2		*1.1*	*0.9*												PA2	8	*1.1*	*1.9*	1.5
PS26	7	3.7	*0.5*	2.7	1.4							PT5	6				PT1			0.7	1.3
PS29	14	14.1	*0.6*	8.9	1.5												PT2			0.6	1.3
PS30	9	3		6.6	2												PT3	14		0.6	1.4
PS32	8	*1.8*			*0.9*												PT4	8		1	1.7
PS33	10	2.9			1												PT6	14		1.1	2
PS34	14	2.1		4	1.7												PT7	8	*0.5*	*0.9*	1.2
PS35	14	9.6	*0.6*	7.8	1.4													7			
PS36	11	*1.6*		*0.4*														11			
PS37	14	10.2	*0.8*	7.2	1.6																
PS38	11	2.8		*1.7*	1.1																
PS41	10	2		*1.3*	*0.9*																

^a^ Number in italics: <LOQ (OTA 2 ng/mL, OTB and STER 1 ng/mL). Empty spaces: values < limit of detection (LOD), LODs: OTA and OTB 0.4 ng/mL, STER 0.2 ng/mL. AE: after enzymatic treatment; BE: before enzymatic treatment. PA: ASD. PD: digestive problems. PS: healthy. PT: ADHD.

**Table 4:** Summary of the data obtained for OTA before and after enzymatic treatment.

	Health Status	Gender	*n*	% Positives	Mean ± SD (ng/mL)	Median (ng/mL)	Max Value (ng/mL)
BE	Healthy	Girls	28	89.3	3.27 ± 3.10	2.27	14.07
		Boys	12	100	3.08 ± 1.37	2.50	6.03
		Total	40	92.5	3.21 ± 2.69	2.42	14.07
	Digestive disorder	Girls	15	46.7	1.10 ± 1.07	0.40	3.39
		Boys	15	26.7	0.89 ± 1.47	0.40	6.07
		Total	30	36.7	0.99 ± 1.27	0.40	6.07
AE	Healthy	Girls	28	89.3	3.49 ± 3.15	2.07	11.92
		Boys	12	100	2.82 ± 1.11	2.50	4.69
		Total	40	92.5	3.29 ± 2.70	2.36	11.92
	Digestive disorder	Girls	15	80	2.33 ± 4.75	0.75	19.27
		Boys	15	73.3	1.46 ± 1.48	1.14	5.75
		Total	30	76.7	1.90 ± 3.48	1.04	19.27

AE: after enzymatic treatment. BE: before enzymatic treatment. SD: standard deviation.

**Table 5:** Summary of the data obtained for STER after enzymatic treatment.

Health Status	Gender	*n*	% Positives	Mean ± SD (ng/mL)	Median (ng/mL)	Max Value (ng/mL)
Healthy	Girls	28	96.4	1.43 ± 0.34	1.44	2.51
	Boys	12	100	1.36 ± 0.46	1.26	2.54
	Total	40	97.5	1.41 ± 0.38	1.41	2.54
Digestive Disorders	Boys	15	100	1.51 ± 0.25	1.57	1.91
	Girls	15	100	1.55 ± 0.41	1.48	2.35
	Total	30	100	1.53 ± 0.33	1.54	2.35

SD: standard deviation.

**Table 6:** Chromatographic parameters.

Column	Ascentis Express C18, 2.7 μm, 150 × 2.1 mm (Supelco Analytical, St. Louis, MO, USA) at 45 °C
Mobile Phase	Mixture of A (5 mM ammonium formate and 0.1% formic acid in water) and B (5 mM ammonium formate and 0.1% formic acid in 95:5 methanol/water) in gradient conditions
Flow Rate	0.4 mL/min
Volume of Injection	20 μL

## Data Availability

Data is contained within the article or supplementary material.

## Supplementary Materials

The following are available online at https://www.mdpi.com/2072-6651/13/2/150/s1, Table S1: Examples of the obtained calibration curves. Table S2: Statistical *p*-values on the occurrence of OTA in samples.

## Abbreviations

15-ADON	15-acetyldeoxynivalenol
3-ADON	3-acetyldeoxynivalenol
ACN	Acetonitrile
ADHD	Attention deficit hyperactivity disorder
AFB1	Aflatoxin B1
AFB2	Aflatoxin B2
AFG1	Aflatoxin G1
AFG2	Aflatoxin G2
AFM1	Aflatoxin M1
AFs	Aflatoxins
ASD	Autism spectrum disorder
CI	Confidence interval
DAS	Diacetoxyscirpenol
DOM-1	Deepoxy-deoxynivalenol
DON	Deoxynivalenol
EDTA	Ethylendiaminetetraacetic acid
EMA	European Medicines Agency
ESI	Electrospray ionization
FBs	Fumonisins
FDA	Food and Drug Administration
FUS-X	Fusarenon-X
HT-2	HT-2 toxin
IARC	International Agency for Research on Cancer
LC	Liquid chromatography
LC-MS/MS	Liquid chromatography–mass spectrometry
LOD	Limit of detection
LOQ	Limit of quantification
NEO	Neosolaniol
NIV	Nivalenol
OTA	Ochratoxin A
OTA-d_5_	Ochratoxin A-(*phenyl*-d_5_)
OTB	Ochratoxin B
PAT	Patulin
PBS	Phosphate buffer solution
q	Transition of qualification
Q	Transition of quantification
QqQ	Triple quadrupole
RE	Relative error of the mean
RSD	Relative standard deviation
RT	Retention time
SD	Standard deviation
STER	Sterigmatocystin
T-2	T-2 toxin
TCT	Trichothecenes
ZEA	Zearalenone
