# Supplementary material for: Assessment of Exposure to Mycotoxins in Spanish Children through the Analysis of Their Levels in Plasma Samples
Source: Toxins (Basel). 2021 Feb 15;13(2):150. doi: 10.3390/toxins13020150 (PMC7919644; doi:10.3390/toxins13020150)
Supplement: Supplementary file 1 [file toxins-13-00150-s001.pdf]

Supplementary Materials: Assessment of Exposure to Mycotoxins in Spanish Children through the Analysis of Their Levels in Plasma Samples

Beatriz Arce-López, Elena Lizarraga, Reyes López de Mesa and Elena González-Peñas

Table S1. Calibration curves for each of the analysed mycotoxins before and after enzymatic treatment.

	Mycotoxin	Range (ng/mL)	LOD (ng/mL)	Before Enzymatic Treatment			After Enzymatic Treatment		
				R^2	Calibration Curve	Weight	R^2	Calibration Curve	Weight
Group I	DOM-1	6.0-180.0	1.35	0.9991	y=18.53x − 37.58	None	0.9983	y=17.23x + 2.36	None
	AFG2	0.8-24.0	0.35	0.9967	y=118.90 + 76.61	None	0.9987	y=155.31 − 59.05	None
	AFM1	0.8-24.0	0.18	0.9973	y=89.25x + 58.70	None	0.9964	y=102.06x + 0.58	None
	AFG1	0.3-9.0	0.07	0.9914	y=265.28x + 156.33	None	0.9984	y=348.78x − 14.64	None
	AFB2	0.3-9.0	0.07	0.9969	y=275.95x + 69.75	None	0.9974	y=319.86x − 46.42	None
	AFB1	0.2-6.0	0.04	0.9962	y=521.83x + 87.81	None	0.9990	y=624.90x − 51.15	None
	HT-2	6.0-180.0	2.70	0.9978	y=13.73x + 47.75	None	0.9979	y=11.56x − 19.68	None
	OTB	1.0-30.0	0.40	0.9992	y=183.82x − 62.10	1/x	0.9909	y=130.58x + 98.58	1/x
	T-2	1.0-30.0	0.20	0.9970	y=87.67x + 96.80	None	0.9990	y=73.80x − 11.32	None
	ZEA	4.0-120.0	1.80	0.9935	y=21.80x + 136.51	None	0.9964	y=21.85x − 90.44	None
	OTA-d_5	2.0-60.0	0.40	0.9956	y=56.46x − 11.59	1/x	0.9982	y=40.39x − 31.16	None
	STER	1.0-30.0	0.20	0.9970	y=118.10x + 94.60	None	0.9930	y=136.82x − 74.18	None
Group II	NIV	20.4-612.0	9.10	0.9969	y=1.39x − 9.45	None	0.9941	y=1.21x − 27.45	None
	DON	8.72-361.6	1.94	0.9994	y=6.47x − 3.89	None	0.9968	y=6.31x − 8.69	None
	FUS-X	7.0-210.0	1.95	0.9984	y=4.64x + 8.89	None	0.9944	y=3.51x − 8.98	None
	NEO	0.8-24.0	0.18	0.9944	y=57.02x + 27.60	None	0.9952	y=42.86x − 10.85	None
	3-ADON	1.75-52.5	0.70	0.9962	y=20.48x + 5.92	None	0.9905	y=15.91x + 24.90	None
	15-ADON	2.72-81.6	1.20	0.9990	y=21.82x − 14.97	None	0.9961	y=16.84x − 23.71	None
	DAS	0.7-21.0	0.15	0.9982	y=111.29x + 15.60	None	0.9970	y=67.75x + 29.55	None

LOD: limit of detection.

Table S2. Statistical *p*-values on the occurrence of OTA in samples.

AE	PD	T						0.04*			1.8E-4*			
		B										0.84		
		G											0.84	
	PS	T			0.82									1.8E-4*
		B							0.99					
		G								0.99				
BE	PD	T			3.5E-7*									0.04*
		B												
		G												
	PS	T						3.5E-7*			0.82			
		B	0.62			0.26								
		G		0.62			0.26							
			G	B	T	G	B	T	G	B	T	G	B	T
			PS			PD			PS			PD		
			BE						AE					

*: statistical significance (p < 0.05, 95% CI). AE: after enzymatic treatment. BE: before enzymatic treatment. B: boys. G: girls. PS: healthy children. PD: children with digestive disorders. T: total.
